# Quantitative Mass Spectrometry‐Based Biodistribution of Monoclonal Antibodies: An Alternative to Radio‐Biodistribution

**DOI:** 10.1002/advs.202520265

**Published:** 2026-04-16

**Authors:** Domenico Ravazza, Sheila Dakhel Plaza, Samuele Cazzamalli, Andrea Ciamarone, Andrea Galbiati, Riccardo Stucchi, Frederik Peissert, Emanuele Puca, Dario Neri, Ettore Gilardoni

**Affiliations:** ^1^ Philochem AG Otelfingen Switzerland; ^2^ Philogen S.P.A Siena Italy; ^3^ Department of Applied Biosciences Swiss Federal Institute of Technology Zurich Switzerland

**Keywords:** biodistribution, liquid chromatography, mAbs, mass spectrometry, stable isotopically labelled internal standard

## Abstract

Monoclonal antibodies (mAbs) are excellent tools for generating targeted therapies for cancer treatment, and an early assessment of their biodistribution properties is essential in the discovery process. Traditionally, radiolabeling methods are widely used, but they require structural alterations of the antibody, radiation exposure, and specialized infrastructure. In this work, we present the implementation of a non‐radioactive Mass Spectrometry (MS)‐based method to assess the ex vivo quantitative biodistribution of tumor‐targeting mAbs directed against the tumor extracellular matrix. By combining protein A purification with a stable isotopically labeled standard, we obtained a versatile method that can be easily transferred to different analytes. The methodology was orthogonally validated by direct comparison against radiolabel‐based biodistribution studies, demonstrating high reliability and accuracy.

## Graphical abstract

Monoclonal antibodies are powerful anticancer therapeutics targeting tumor‐associated antigens. Mass spectrometry enables precise and accurate quantification of mAbs in tumor plasma and healthy organs. Unlike ligand binding assay and radioactivity, mass spectrometry does not suffer from matrix effects and labelling artefacts while facilitating method transfer across molecules.

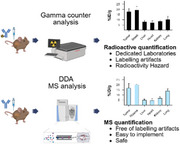

## 1 Introduction

*Ex vivo* tissues quantification is an essential requirement to study the pharmacokinetics (PK), pharmacodynamics (PD), and target engagement of innovative biopharmaceuticals, both at the preclinical and clinical levels [1]. Historically, ligand binding assays (LBA) such as Enzyme‐Linked Immunosorbent Assay (ELISA), have been the method of choice to quantify biopharmaceuticals [2, 3, 4]. However, developing such methods is usually time‐consuming, expensive [5], and highly analyte‐specific [6]. LBA also requires high‐quality reagents to ensure optimal selectivity for the unambiguous identification of the target analyte [7], and they often suffer from significant matrix effects, limiting their transferability across tissues [8]. In fact, most LBA‐based methods are routinely applied only to study plasma PK.

Radiolabeling of monoclonal antibodies (mAbs) [9] offers the possibility to track them in different matrices with good sensitivity, and it is often used for the preclinical investigation of mAbs PK [10, 11, 12]. Depending on the radiotracer, tissue accumulation can be measured either via imaging technique (e.g., immune‐PET [13] or SPECT [14]) or *ex vivo*, by measuring the radioactive uptake in each tissue using a gamma‐counter [15]. Nonetheless, the requirements of dedicated laboratories and the limitations of radiation exposure restrict the widespread applicability of this methodology. Additionally, there is evidence that the radiolabeling procedure can compromise the stability of the mAbs or alter the biodistribution profile [9].

Liquid chromatography mass spectrometry (LC‐MS) methods for protein quantification in biological matrices have rapidly evolved in the last 15 years and now represent compelling alternatives to the LBA and radiolabeling techniques [16, 17, 18, 19]. Owing to their high selectivity, accuracy, and precision, LC‐MS approaches are routinely applied in PK studies [20, 21]. However, methodologies reported in the literature vary considerably. Since the sensitivity of mass spectrometers is greater for peptides than for intact proteins [22], most workflows include proteolytic digestion followed by peptide‐level analysis [19]. To increase sensitivity, various enrichment strategies have been employed, either prior to proteolytic digestion (e.g., Protein A [23], cognate antigen [24, 25], Anti‐Fc [26, 27, 28], Anti‐idiotypic reagents [29]) or at the peptide level (e.g., anti‐peptide antibody [30, 31], multi‐dimensional separation [32]). The selection of the Internal Standard (IS) is also critical for ensuring accurate quantification [33]. Stable‐Isotope‐Labelled (SIL) peptides are commonly spiked in prior to LC‐MS analysis, also due to their wide availability and low cost [34]. To account for digestion variability, the use of extended SIL peptides [35] or of homologous antibodies has also been explored [24]. However, the ideal internal standard for MS‐based applications would be the full SIL protein as it completely retains the physicochemical properties of the corresponding analyte while remaining distinguishable by the mass spectrometer [36]. Nevertheless, their use is limited due to historically high production costs and restricted availability [20].

Most published works on antibody quantification focus on a single biological matrix (i.e., serum or plasma), with only a few reporting analysis in multiple matrices [28, 37, 38, 39]. To the best of our knowledge, a generic and easily implementable method to accurately measure antibody concentrations in different tissues of tumor‐bearing mice has not yet been described. Here, we report the implementation of a mass spectrometry‐based method to generate quantitative biodistribution data of mAbs in tumor, plasma, and healthy organs in preclinical models. The accuracy and reliability of our method were confirmed by comparing the data against two different orthogonal radioactivity‐based biodistribution experiments.

## 2 Results

### 2.1 Internal Standard Identification

**FIGURE 1:**
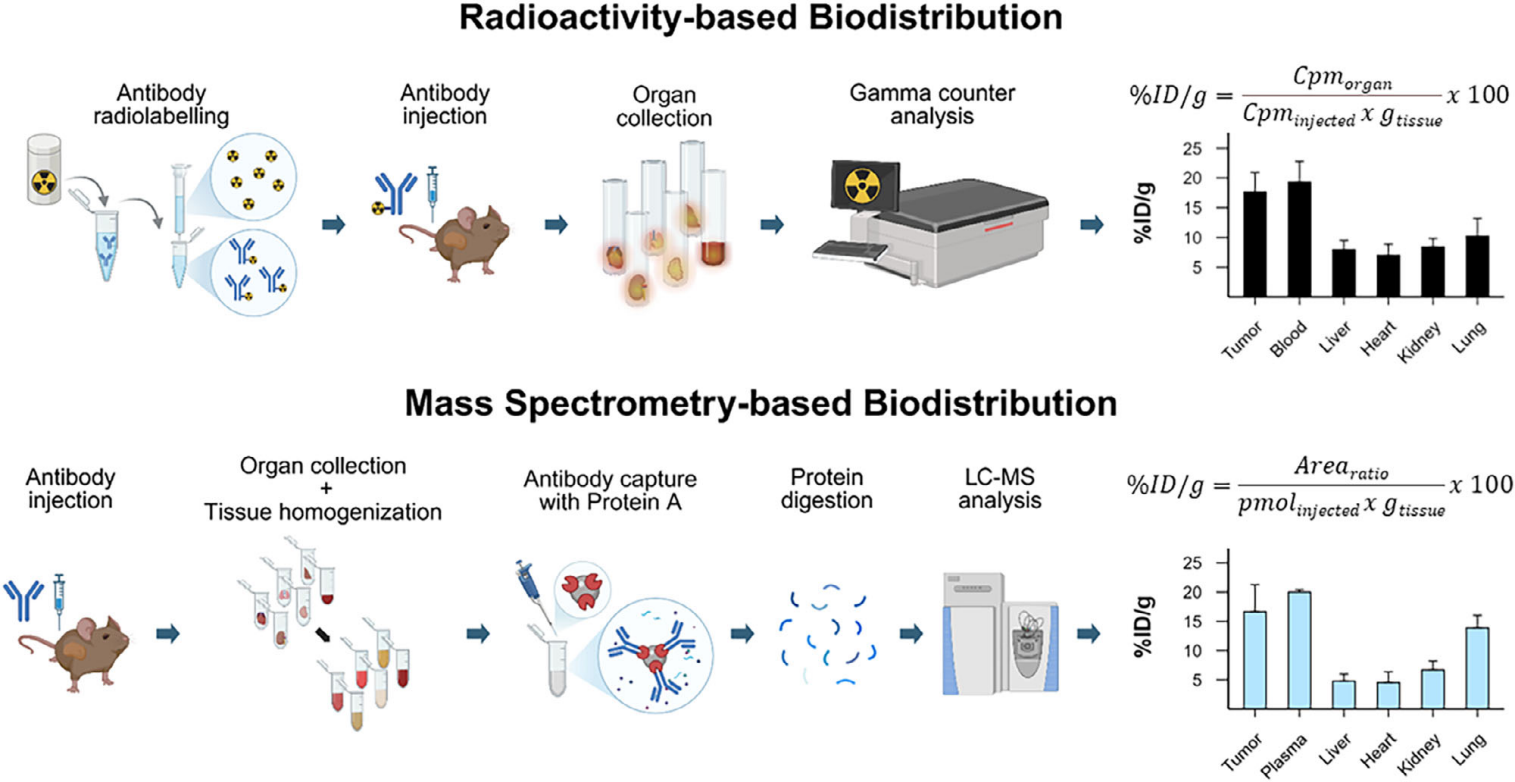
Schematic workflow of the experimental design and sample preparation procedure. For the radioactivity‐based biodistribution (upper panel) tumor‐bearing mice are injected with the radiolabeled antibody, and at the desired time point, organs are collected and analyzed with a gamma counter. For the mass spectrometry‐based biodistribution (lower panel), tumor‐bearing mice are injected with the antibody and at the desired time point organs are collected and homogenized; antibodies are then enriched with Protein A and finally digested; the resulting peptides are analyzed by means of LC‐MS.

Mass spectrometry offers the sensitivity required to detect and quantify large biopharmaceuticals. However, due to their large size and heterogeneity, an optimized sample preparation workflow is essential to ensure efficient recovery, digestion, and analysis while maintaining quantitative accuracy. For this reason, we have implemented a workflow based on Protein A affinity chromatography to selectively enrich the analyte and reduce matrix interference. Enriched proteins were then subjected to reduction, alkylation, and digestion under controlled conditions commonly used for monoclonal antibody peptide mapping [40], minimizing preparation‐induced artifacts. The resulting peptides were analyzed via LC‐MS (Figure 1). Absolute quantification is achieved using an internal standard (IS) to compensate for the variabilities introduced during antibody enrichment, protein digestion, and LC‐MS analysis.

**FIGURE 2:**
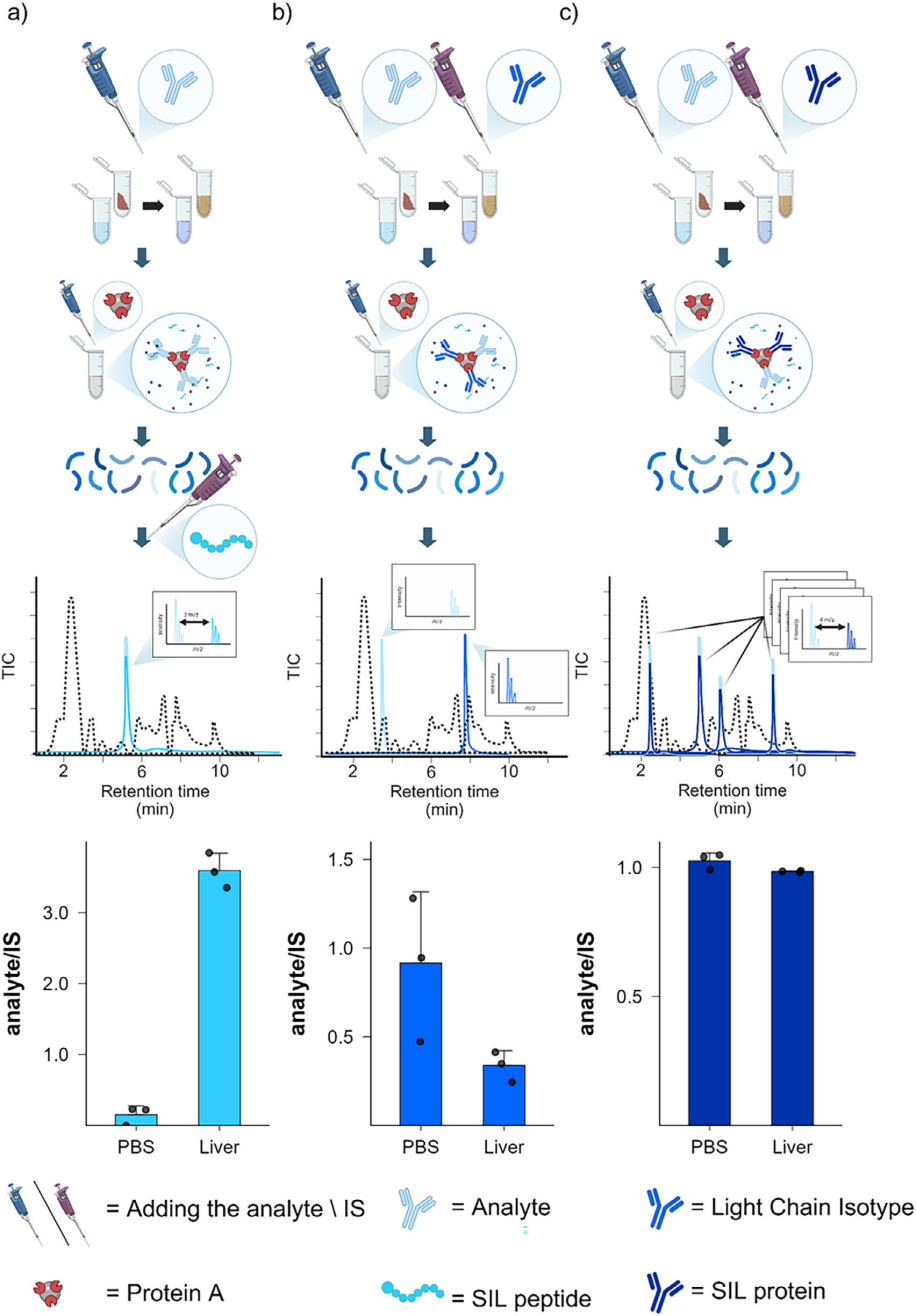
Comparison of analyte to IS ratios measured in blank matrices. The analyte is added in equimolar amount with the (a) SIL peptide in PBS and Liver; (b) Light Chain Isotype in PBS and Liver; (c) SIL Protein in PBS and Liver. Error bars represent the standard deviation.

To find the most suitable IS for our method, we tested three candidates using the L19 antibody (specific to the extra domain B of fibronectin) as a model: (i) a stable isotope‐labeled (SIL) peptide, (ii) a homologous antibody with a different light chain isotype, and (iii) a full‐length SIL L19 antibody (Figure 2). The three IS were spiked at an equimolar amount with the L19 antibody analyte (10 pmol) in buffer (i.e., PBS) or blank tissue (i.e., liver). The IS antibodies were added before the homogenization step, whereas the SIL peptide was added after the on‐beads digestion step (Figure 2).

The SIL peptide, ALPAPIEK (derived from the heavy chain of L19), was selected for its high signal‐to‐noise ratio and a relatively short sequence length. In the spike‐in experiment, the analyte‐to‐IS ratio deviated from the theoretical value of 1 and differed between the two matrices. These results indicated a significant matrix effect and lack of compensation for variability introduced during enrichment and digestion (Figure 2A and Table S1).

Next, we tested a homologous antibody (i.e., KSF specific to hen‐egg lysozyme) featuring a different light chain than the analyte (i.e., L19 antibody). The IS was added at the beginning of sample preparation to compensate for potential variability arising during the enrichment and digestion steps. Signature peptides that could be uniquely detected and quantified by the mass spectrometer were selected for both KSF and L19. Three and six signature peptides for the analyte and for the IS were identified, respectively. The analyte‐to‐IS ratio was calculated from the average area of the signature peptides. Despite full‐process integration, ratios still deviated from the theoretical value of 1 and varied between the two matrices (Figure 2B and Table S1), likely due to differing peptide recovery or digestion efficiencies between the two antibodies and peptides LC‐MS behavior.

Finally, we tested a labeled SIL version of L19 with [^13^C_6_^15^N_2_]‐lysine and [^13^C_6_^15^N_4_]‐arginine. In this case, the antibody added as IS is the heavy version of the analyte of interest (i.e., heavy isotope incorporation >99.5%; Table S2). After tryptic digestion, forty‐four (44) L19‐derived peptides (with more than six amino acids) with at least one arginine or lysine were generated. This provided a large pool of choice in the identification of the best peptide combination for the calculation of the analyte‐to‐IS ratio. To guarantee that the LC‐MS readout reflects the intact monoclonal antibodies and not Fc‐containing catabolites, or degradation products that could occur during sample preparation, we selected peptides that represent the variable and constant regions of both the heavy and light chains. 11 peptides in total (3 from the light chain and 8 from the heavy chain) were chosen as signature peptides (Table S2 and Figure S1A) to calculate the average light‐to‐heavy ratio (Figure 2C and Table S1). The ratios were reproducible across all antibody regions and closely matched the theoretical ratio of one in both samples. Moreover, these results were confirmed in additional tissues, where the SIL L19 antibody consistently provided accurate and matrix‐independent correction (Figure S1B and Table S1). Based on these findings, the SIL L19 antibody was selected as the internal standard for subsequent *ex vivo* biodistribution experiments.

### 2.2 *Ex Vivo* Quantitative Biodistribution Studies

Biodistribution studies of the fully‐human L19 and F8 monoclonal antibodies in the IgG format were performed in F9 teratocarcinoma‐bearing mice [41, 42]. L19 and F8 bind selectively to the extra‐domain B (EDB) and A (EDA) of oncofetal fibronectin, which are highly expressed in the microenvironment of different cancer types, but undetectable in virtually all healthy tissues [43]. One nmol of either L19 or F8 was intravenously administered in the lateral tail vein of tumor‐bearing mice. Animals were euthanized, and organs resected 24 h after the administration of the antibodies.

**FIGURE 3:**
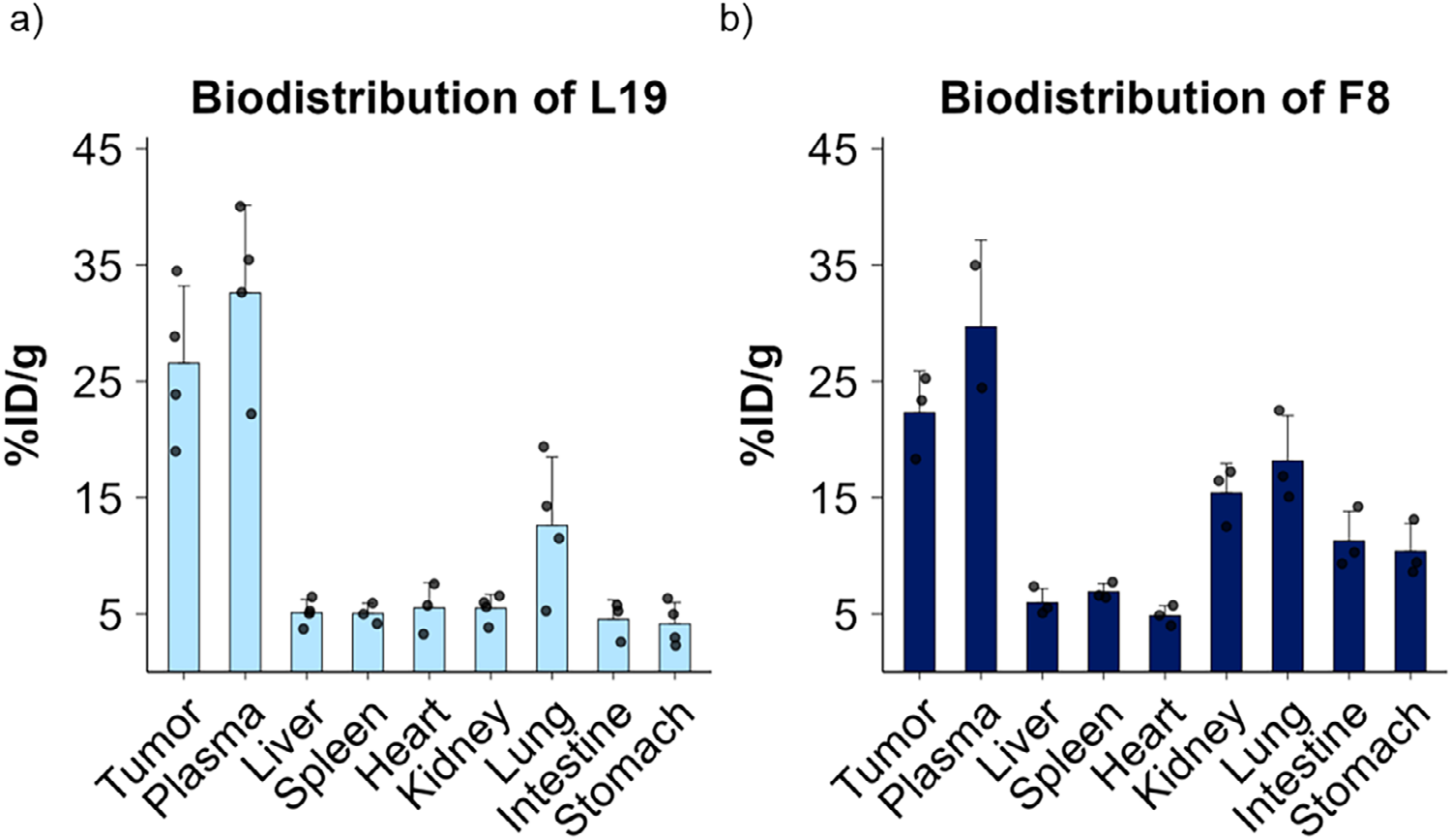
Mass Spectrometry‐based *ex vivo* biodistribution for (a) L19 and (b) F8. Mice bearing subcutaneous F9 tumors were sacrificed 24 h after intravenous administration of 1 nmol of antibody. Error bars represent the standard deviation.

The highest percentage injected dose per gram (%ID/g) value for L19 was found in plasma, consistent with the long circulatory half‐life of IgGs, followed by significant accumulation in the tumor (Figure 3A and Table S4). All other organs exhibited%ID/g values below 6%, except for the lungs. Overall, tumor‐to‐organ ratios exceeded 4 for all organs except plasma and lung (Table S5). The relatively high lung uptake likely reflects residual blood content; in fact, the lungs are known to be highly perfused, and up to 50% of the lung weight is constituted by blood [44]. This influences the absolute value that is measured in this tissue. If residual blood in the tissue is interfering with biodistribution results, blood perfusion prior to organ harvesting can be considered.

A similar biodistribution profile was obtained for the F8 antibody (Figure 3B, Tables S6 and S3), with the highest% ID/g recorded in tumor (∼23%) and in plasma (∼30%), and with comparable tumor‐to‐organ ratios (Table S7).

### 2.3 *Ex Vivo* Quantitative Biodistribution With Radiolabeled Antibodies

*Ex vivo* biodistribution methods based on the administration of radiolabeled antibody preparations remain the gold‐standard methodology for determining tumor‐targeting properties of antibodies at the pre‐clinical level. To validate the results obtained with the mass spectrometry approach, we performed biodistribution studies of ^89^Zr‐ and ^125^I‐labelled IgG (L19). ^89^Zr is a positron‐emitting radionuclide [45] with a radioactive half‐life of 78.4 h, compatible with the biological half‐life of antibodies [46]. It is routinely used for immune‐PET studies in animal models and human [47], and *ex vivo* gamma‐biodistribution in animal models [48]. ^125^I has a radioactive half‐life of 59.3 days and decays via electron capture followed by gamma‐decay [49]. It is commonly used for labelling antibodies in radioimmunoassay [9, 50] or to study the biodistribution of antibodies in animal models [15, 51]. The antibody was first bioconjugated with p‐isothiocyanatobenzyl‐desferrioxamine (DFO) chelator to enable ^89^Zr radiolabeling [52, 53]. To preserve antigen‐binding activity, we targeted a chelator‐to‐antibody ratio (CAR) of 1–1.5 (Figure S3A), as higher CARs have been associated with impaired binding affinity [54]. This was confirmed by performing surface plasmon resonance binding experiments on an EDB‐coated chip with L19, either with or without the DFO conjugation. The DFO‐conjugated and unconjugated L19 exhibited comparable dissociation constant (K_D_) values (L19 K_D_ = 4.9 ± 2.27 nM; L19‐DFO K_D_ = 9.19 ± 3.39 nM) (Figure S3B). L19‐DFO was subsequently radiolabeled with ^89^Zr. By contrast, ^125^I‐L19 was directly generated by radio iodination with Na^125^I salt in the presence of chloramine T.

**FIGURE 4:**
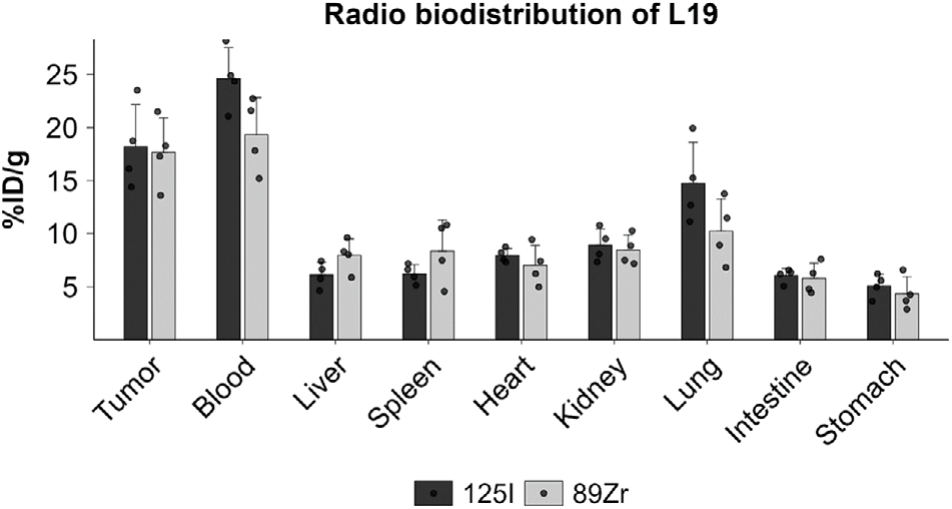
Radioactivity‐based *ex vivo* biodistribution data of L19 performed with ^125^I and ^89^Zr. Mice bearing subcutaneous F9 tumors were sacrificed 24 h after intravenous administration of 200 µg of antibody labelled with 1.8 MBq of ^125^I or 3.1 MBq of ^89^Zr. Error bars represent the standard deviation.

Both ^89^Zr‐L19 and ^125^I‐L19 exhibited similar biodistribution profiles when assessed 24 h post intravenous administration in tumor‐bearing mice (Figure 4, Tables S8 and S9). In line with what was observed with the MS‐based approach (Figure 3), the highest%ID/g values were found in blood and the tumor, with a relatively clean profile across all other healthy organs. Notably, the uptake in the lungs was higher than for other organs, in line with what was seen in the MS‐based biodistribution.

### 2.4 Validation of Mass Spectrometry‐Based Analysis

**FIGURE 5:**
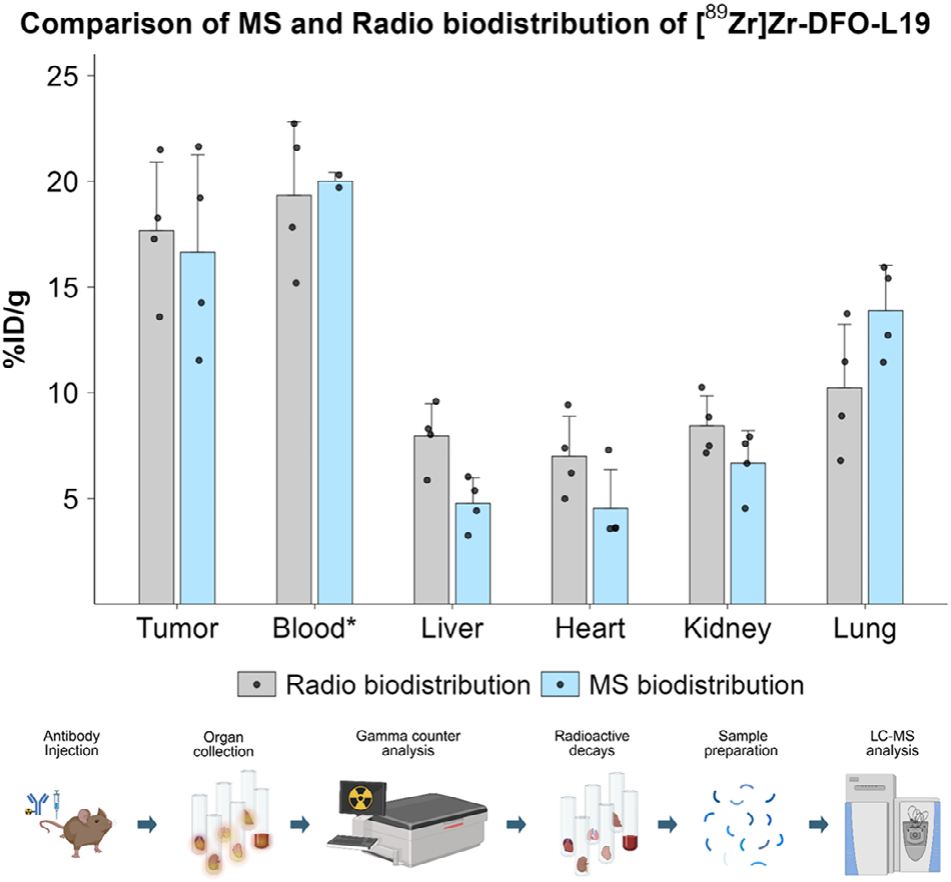
Comparison of Mass spectrometry‐ and radioactivity‐based *ex vivo* biodistribution of L19. Mice bearing F9 tumors were sacrificed 24 h after intravenous administration of 200 µg of antibody labelled with 3.1 MBq of ^89^Zr. Mass Spectrometry analysis was carried out on the same sample, after complete radioactivity decay. A statistically significant difference between the absolute value measured by the two methodologies was observed only in the liver (*p* < 0.05, multiple *t*‐test). *For MS biodistribution plasma was used instead of blood. Error bars represent the standard deviation.

Ex vivo mass spectrometry (Figure 3A) and radio (Figure 4) biodistributions of L19 IgG showed similar trends, with higher tumor and plasma values compared to other healthy organs. To directly compare the two techniques under the same biological conditions, we leveraged the relatively short half‐life of ^89^Zr (i.e., 78.4 h) and collected selective organs from the radioactive experiment following complete radioactivity decay. The same tissues were processed and re‐analyzed with the mass spectrometry‐based methodology. Figure 5 (Table S10) shows a side‐by‐side comparison of the L19 biodistribution data obtained on the same samples with either ^89^Zr or mass spectrometry. In this matched analysis, the mass spectrometry data closely aligned with the data obtained with the radiolabeled antibody, both in terms of relative and absolute uptake in the analyzed organs (Table S11).

## 3 Conclusion

Determining the biodistribution properties of monoclonal antibodies, and in general biopharmaceutical products, is an important step in the discovery and preclinical characterization of such molecules. In this work, we aimed at implementing a mass spectrometry‐based workflow for the quantification of monoclonal antibodies in *ex vivo* biodistribution experiments. After tissue homogenization, sample complexity is decreased via an affinity purification step performed with Protein A, a toxin produced by Staphylococcus aureus that recognizes and binds to the Fc region of IgGs. A SIL version of the analyte was used as IS, allowing for a proper analytic variability correction both at the sample preparation and LC‐MS analysis level. For a long period of time, SIL proteins have been expensive to produce. However, the development of a depleted medium specifically designed to produce monoclonal antibodies has reversed the situation, making it easier and cheaper than synthesizing a single SIL peptide.

The mass spectrometry method presented here was applied to study L19 and F8, two tumor‐targeting antibodies that are specific to very abundant and stable antigens, expressed in the tumor stroma, and are being used in the clinic to deliver cytokines and cytotoxic drugs [55, 56]. Radiobiodistribution with ^125^I and ^89^Zr served as reference methods. Although radiolabeling is widely used for quantitative biodistribution studies, it can in some cases, introduce artifacts. For example, direct radioiodination may modify tyrosine residues within or near antibody CDRs and thereby reduce immunoreactivity [57]. In contrast, ^89^Zr labeling relies on prior conjugation of a chelator (DFO) to the antibody and subsequent radiometal complexation. In the present work, SPR measurements confirmed unchanged L19 affinity after DFO conjugation (Figure S3), and the strong concordance between the ^125^I‐ and ^89^Zr‐based biodistribution profiles, obtained using distinct labeling chemistries, suggests that radiolabeling did not measurably alter the in vivo behavior of L19 under our experimental conditions. Therefore, radiotracer biodistribution provides an appropriate benchmark for the MS‐based quantification. To the best of our knowledge, this is the first report where an MS‐based workflow has been applied to determine the *ex vivo* biodistribution of a monoclonal antibody in tumor‐bearing mice and has been orthogonally validated with radioactivity. These findings highlight the potential of mass spectrometry as a powerful technique for PK characterization of biologics. MS not only provides a safer and more accessible analytical platform but also enables direct quantification of the unmodified therapeutic molecule. For this reason, a more accurate assessment of the in vivo behavior is achieved, avoiding the introduction of any chemical or radiolabeling modification.

When moving into clinical settings, radiolabeling enables real‐time monitoring of the antibody biodistribution via imaging techniques. While impractical for whole‐body biodistribution studies, where biopsies of every tissue would be necessary, mass spectrometry can still be used to monitor antibody plasma pharmacokinetics or tumor accumulation, and facilitate the implementation of Therapeutic Drug Monitoring of monoclonal antibodies and other biopharmaceuticals in clinical settings [58], reducing the complexity of developing specific reagents normally required for ligand binding assays. The use of well‐established antibodies (i.e., L19 and F8) limits this work to the validation of the methodology. However, the good performance and reliability of the results obtained give no cause for concern about the method's ability to analyze any discovery‐phase IgG in dedicated biodistribution experiments. Future development would enable the expansion and implementation of the work to a broader range of biotherapeutic formats, including antibody fragments, immunocytokines, and antibody‐drug conjugates (ADCs). Doing so, mass spectrometry‐based methodologies would increase the speed of new biological entities discovery and facilitate the selection of the ideal clinical candidate.

## 4 Experimental Section/Methods

### 4.1 Cell Lines

F9 murine teratocarcinoma (RRID:CVCL_0258) and Chinese Hamster Ovary cell lines (CHO, RRID:CVCL_0213) were purchased from American Type Culture Collection (ATCC). Stable cell lines for the production of L19 IgG (CHO‐L19) and F8 IgG (CHO‐F8) were available in‐house. CHO stable cell lines were grown in Power‐CHO serum‐free medium (Lonza) supplemented with 4 mM L‐glutamine (Lonza) in a shaking incubator at 37°C and moved to 31°C for production. CHO cells for transient gene expression (TGE) were grown in Power‐CHO serum‐free medium (Lonza) supplemented with 4 mM L‐glutamine (Lonza) in a shaking incubator at 37°C. For production, the media was changed to ProCHO (Lonza) and incubated at 31°C. F9 cells were grown on 0.1% gelatin‐coated flasks (Sigma Aldrich) in Dulbecco″s Modified Eagle Medium (DMEM,Gibco) + 10% Fetal Bovine Serum (FBS, Gibco) for no longer than 10 passages. Date of purchase of cell lines is missing; all cell lines are contaminant‐free.

### 4.2 Reagents

If not differently stated, all reagents and solvents were purchased from Sigma‐Aldrich, VWR, Combi‐Blocks, CheMatech or Eurisotope and used as supplied. L19IgG, F8 IgG, and KSF IgG were produced in‐house, and their sequences are reported in the supplementary information.

### 4.3 SIL Peptide Synthesis

[^13^C_3_‐^15^N_1_] ALPAPIEK SIL peptide was synthesized by means of solid phase peptide synthesis (SPPS) and in solution synthesis as reported in the supplementary information. Briefly, amino acids from lysine to leucine were coupled via FMOC chemistry using a Wang resin as support. The peptide was cleaved from the resin in acidic conditions and coupled to ^13^C_3_‐^15^N_1_‐Fmoc‐Alanine. After deprotection, the [^13^C_3_‐^15^N_1_] ALPAPIEK SIL peptide was obtained.

### 4.4 Antibodies Production

Fully human monoclonal antibodies produced in‐house were used for this study. L19 and F8 IgG were expressed using CHO stable cell lines. KSF IgG was produced by PEI‐induced TGE in CHO cells using the expression vector pMM137‐KSF (available in‐house). All antibodies were purified by Protein A affinity chromatography. Sodium Dodecyl Sulfate‐Polyacrylamide Gel Electrophoresis (SDS‐PAGE), Size Exclusion Chromatography (SEC), and intact protein mass spectrometry analyses were used as quality control. Quality control data are available in the supplementary information.

### 4.5 SIL Monoclonal Antibodies

L19 and F8 stable CHO cell lines were grown in CHO‐TF for SILAC (Sartorius) supplemented with stable isotopically labelled arginine and lysine (i.e., [^13^C_6_, ^15^N_4_]L‐arginine, Thermo Fisher; [^13^C_6_, ^15^N_2_]L‐lysine, Sigma‐Aldrich), according to manufacturer instructions. Antibodies were purified by Protein A affinity chromatography. SDS‐PAGE, SEC, and intact protein mass spectrometry analyses were used as quality control. SIL amino acids incorporation efficiency was evaluated by peptide mapping analysis. Quality control data are available in the supplementary information.

### 4.6 Surface Plasmon Resonance Analysis

Kinetic parameters of the binding interaction of L19 with EDB were measured by Surface Plasmon Resonance on a BiacoreX100 (Cytiva, RRID:SCR_025586). Recombinant EDB was immobilized on a CM5 sensor chip at a density of 1500 RU using standard amine coupling reaction as per manufacturer´s instructions. mAbs samples were analyzed in serial two‐fold dilutions at a flow rate of 10 µL/min using HBS‐EP+ as running buffer and 10 mM HCl as regeneration buffer. Binding curves were analyzed using BIAevaluation3.2 software (Cytiva, RRID:SCR_015936).

### 4.7 DFO Bioconjugation

L19 was buffer exchanged into NaHCO_3_ 0.1 M pH 9 at a final concentration of 4 mg mL^−1^. p‐SCN‐Bn‐DFO (CheMatech) was dissolved in DMSO, and a 10‐fold molar excess was added to the antibody solution. The bioconjugation reaction was carried out at 37°C for 1 h with gentle agitation and purified with a PD10 desalting column (Cytiva), using HEPES 0.5 M as buffer. Bioconjugation efficiency and chelate‐to‐antibody ratio were evaluated by intact protein mass spectrometry analysis (supplementary information).

### 4.8 Animals Models

All animal experiments were conducted in accordance with Swiss animal welfare laws and regulations under the license number ZH006/2021 granted by the Veterinäramt des Kantons Zurich. 15 million F9 cells (resuspended in 150 µL of HBSS Buffer) were subcutaneously injected in the left flank of 129/sv female mice (Janvier, 6‐8 weeks old, RRID:MGI:2161069). After tumor reached a volume of approximately 100 mm^3^ mice were randomized based on tumor volume in homogeneous group before mAbs injection.

### 4.9 *Ex Vivo* Biodistribution

144 µg (1 nmol) of L19 or F8 were injected into the lateral tail vein of tumor‐bearing mice (*n* = 4). 24 h after the injection mice were euthanized by CO_2_ inhalation, organs harvested, snap frozen with liquid nitrogen, and stored at −80°C until analysis. Blood was collected in heparin tubes, centrifuged at 21 000 *g* for 15 min, and plasma recovered and stored at −80°C.

### 4.10 Sample Preparation for LC‐MS Analysis

Tissues (20 mg) were resuspended in 800 µl of lysis buffer (0.25% sodium deoxycholate, 1 mM Ethylenediaminetetraacetic acid (EDTA), 0.5% Igepal, protease inhibitor (Roche) in PBS pH 7.4). Tissues were then homogenized with a tissue lyser (TissueLyser II, QIAGEN, RRID: SCR_018623) for 30 min at 30 Hz. After homogenization, lysates were centrifuged at 21 000 *g* for 30 min. Supernatants were incubated with BSA‐blocked protein A magnetic beads (Merck) for 2 h under rotation at 4°C. Beads were recovered and washed with lysis buffer, buffer A (150 mM NaCl, 20 mM TrisHCl pH 7.5) and buffer B (400 mM NaCl, 20 mM Tris HCl pH 7.5). Beads were resuspended in digestion buffer (50 mM Tris‐HCl, 1 mM CaCl_2,_ 1 M Urea, pH 8), cystines were reduced at 65°C for 45 min with Tris(2‐carboxyethyl) phosphine (TCEP) (5 mM) and carbamidomethylated at room temperature in the dark for 45 min with iodoacetamide (IAA) (10 mM).To prevent over‐alkylation, excess of IAA was quenched with 10 mM cysteine for 30 min. Finally, proteins were digested on‐beads overnight with trypsin (0.5 µg) at 37°C. Tryptic peptides were purified on C_18_ Macrospin columns (Harvard Apparatus) according to the manufacturer's instructions. Eluates were dried at room temperature with a vacuum centrifuge (Eppendorf). Dried samples were finally resuspended in 100 µL of a solution with 3% of ACN and 0.1% of FA before LC‐MS analysis.

### 4.11 LC‐MS Analysis

1 µL of each sample was injected into the LC‐MS system. Chromatographic separation was carried out on an DNV PepMap Neo RSLC column (75 µm x 15 cm, particle size 2 µm, pore size 100 Å, Thermo Fisher) with a gradient program from 100% A (H_2_O, 0.1% FA), 0% B (ACN 0.1% FA) to 65% A, 35% B in 60 min on an Easy nanoLC 1000 (Thermo Fisher) at a flow rate of 300 nL min^−1^. The LC system was coupled to a Q‐Exactive mass spectrometer (Thermo Fisher, RRID:SCR_020565) via a Nano Flex ion source (Thermo Fisher). Ionization was carried out with 2 kV of spray voltage, 250°C of capillary temperature, and 60 S Lens RF level. The mass spectrometer was working in a data‐dependent top 10 acquisition mode with the following parameters: MS1 scan range: from 374.5 to 1425.5 m/z, HCD NCE: 27, Dynamic exclusion: 10 s. MS/MS spectra were processed and analyzed using Proteome Discoverer (PD, Thermo Fisher, version 2.5, RRID:SCR_014477) and Skyline software (MacCoss Lab Software, version 22.2.0.527, RRID:SCR_014080).

### 4.12 Data Analysis for Samples Containing the SIL Peptide

Database searches were performed against the antibody (i.e., L19) reference sequences, using Sequest as a search engine. Carbamidomethylation of cysteines was set as a fixed modification; oxidation of methionine and [^13^C_3_^15^N_1_] alanine were set as variable modifications, and trypsin was set as cleavage specificity, allowing a maximum of 2 missed cleavages. Data filtering was performed using percolator with a 1% False Discovery Rate (FDR). PD results were imported into Skyline and the ALPAPIEK peptide transitions were manually checked, peak areas integrated and finally the light to heavy area ratio was calculated.

### 4.13 Data Analysis for Samples Containing the Light Chain Isotype

Database searches were conducted against the antibody (i.e., L19) and internal standard (i.e., KSF) reference sequences, using Sequest as a search engine. Carbamidomethylation of cysteines was set as a fixed modification; oxidation of methionine was set as a variable modification, and trypsin was set as cleavage specificity, allowing a maximum of 2 missed cleavages. Data filtering was performed using percolator with a 1% FDR. PD results were imported into Skyline and manually inspected. Peaks areas of signature peptides of the analyte and IS were extracted, and their ratio was calculated.

### 4.14 Data Analysis for Samples Containing the SIL Antibody

Database searches were performed against the antibodies (i.e., L19 or F8) reference sequences, using Sequest as a search engine. Carbamidomethylation of cysteines was set as a fixed modification, oxidation of methionine, [^13^C_6_^15^N_2_] lysine, and [^13^C_6_^15^N_4_] arginine were set as variable modifications, and trypsin was set as cleavage specificity allowing a maximum of 2 missed cleavages. Data filtering was performed using percolator with a 1% FDR. PD results were imported to Skyline and manually inspected. Peak areas of signature peptides of the analyte and IS were extracted and their ratio calculated. For biodistribution samples, the average ratios (analyte/IS) of each sample was compensated for the matrix effect using a single concentration external calibration point and corrected by the total weight of the tissue analyzed to obtain the percentage of injected dose per gram (% ID/g).

### 4.15 *Ex Vivo* Radio Biodistribution

L19‐DFO was radiolabeled with ^89^Zr(oxalate)_2_ by established methods [52]. Briefly, ^89^Zr(oxalate)_2_ was neutralized with 2 M Na_2_CO_3_. The neutralized solution was then added to the antibody, and the labelling reaction was carried out for 1 h at room temperature with gentle rotation and purified using a PD10 desalting column (Cytiva) with PBS as buffer. L19 IgG was radio iodinated with ^125^I and chloramine T and purified using a PD10 desalting column (Cytiva) with PBS as buffer. 200 µg of radiolabeled antibody (3.1 MBq of ^89^Zr, 1.8 MBq of ^125^I) were injected into the tail vein of F9 tumor bearing mice (*n* = 4). 24 h post‐injection mice were euthanized by CO_2_ asphyxiation, organs collected, weighed, and radioactivity measured using a Packard Cobra gamma counter (Packard, Meriden, CT, USA, RRID: SCR_018609). Values are given as the percentage of injected dose per gram (%ID/g).

### 4.16 Statistical analysis

Data were analyzed using R (version 4.3.0, RRID: SCR_000432). Statistical significance between groups was evaluated with a two‐sided homoscedastic multiple *t*‐test.

## Conflicts of Interest

The authors declare no conflicts of interest.

## Supporting information




**Supporting File**: advs75337‐sup‐0001‐SuppMat.docx.

## Data Availability

The data that support the findings of this study are available from the corresponding author upon reasonable request.
