# Supplementary material for: Quantitative Mass Spectrometry‐Based Biodistribution of Monoclonal Antibodies: An Alternative to Radio‐Biodistribution
Source: Adv Sci (Weinh). 2026 Apr 16;13(38):e20265. doi: 10.1002/advs.202520265 (PMC13335672; doi:10.1002/advs.202520265)
Supplement: Supplementary file 1 — Supporting File: advs75337‐sup‐0001‐SuppMat.docx. [file ADVS-13-e20265-s001.docx]

Supporting Information

Quantitative mass spectrometry-based biodistribution of monoclonal antibodies: an alternative to radio-biodistribution

*Domenico Ravazza, Sheila Dakhel Plaza, Samuele Cazzamalli, Andrea Ciamarone, Andrea Galbiati, Riccardo Stucchi, Frederik Peissert, Emanuele Puca, Dario Neri*, Ettore Gilardoni**

**List of abbreviations**

ACN: Acetonitrile

LC-MS: Liquid Chromatography – Mass Spectrometry

HRMS: High Resolution Mass Spectrometry

TCEP: Tris(2-carboxyethyl)phosphine

IAA: Iodoacetamide

SIL: Stable Isotopically Labeled

FA: Formic Acid

DMF: dimethylformamide

HATU: Hexafluorophosphate Azabenzotriazole Tetramethyl Uronium

DCC: N,N′-Dicyclohexylcarbodiimide

DIPEA: N,N-Diisopropylethylamine

TIPS: Triisopropyl silane

TFA: Tri Fluoroacetic Acid

HPLC: High Performance Liquid Chromatography

CID: Collision Induced Dissociation

RT: Room Temperature

SPPS: Solid Phase Peptide Synthesis

RP-HPLC: Reverse Phase High Performance Liquid Chromatography

SDS-PAGE: Sodium dodecyl sulfate–polyacrylamide gel electrophoresis

SEC: Size Exclusion Chromatography

PD: Proteome Discoverer

**Materials and methods**

General Procedures

Liquid Chromatography-Mass Spectrometry (LC-MS) spectra presented were recorded on an Agilent 6100 Series Single Quadrupole MS system combined with an Agilent 1200 Series LC, (RRID:SCR_018037) using an InfinityLab Poroshell 120 EC-C18 Column, (2.7 μm, 4.6 × 50 mm) at a flow rate of 0.8 mL/min, 10% ACN in 0.1% aq. HCOOH to 100% ACN in 5 min.

Reversed-phase high-pressure liquid chromatography (RP-HPLC) was performed on an Agilent 1200 Series RP-HPLC with PDA UV detector, using a Synergi 10μm, MAX-RP 80Å 10 × 250 mm C18 column at a flow rate of 5 mL/min with linear gradients of solvents A and B (A = Millipore water with 0.1% TFA, B = ACN with 0.1% TFA) or with a ThermoFisher UltiMate 3000 (RRID:SCR_019840) with PDA UV detector, using a Hypersil Prep HS phenyl column 5 µm, 150 mm at a flow rate of 30 mL/min with linear gradients of solvents A and B (A = Millipore water with 0.1% TFA, B = ACN with 0.1% TFA).

SEC chromatography was performed using an Äkta (GE Healthcare, RRID:SCR_019958) equipped with a Superdex 200 10/300GL column (GE Healthcare) using PBS as mobile phase at a flow rate of 0.75 mL/min. Proteins were detected by an UV detector at a wavelength of 280 nm.

SDS-PAGE was performed by incubating protein samples (0.2-0.3 mg/mL) in PBS and mixed with either reducing or nonreducing loading buffer. Samples were denatured for 5 min at 95 °C and loaded on NuPAGE 4-12% Bis-Tris Gel (Novex™by Life Technologies). MES NuPAGE (Novex™by Life Technologies) was used as running buffer and electrophoresis was performed at 180 V, 110 mA for 40 minutes. Gel was rinsed with deionized water and stained in Coomassie blue for 15-20 min on an orbital shaker. Staining solution was discarded, and the gel was immersed in destaining solution (acetic acid/methanol/water 1:3:6) on an orbital shaker until sufficient discoloration was observed.

*[^13^C_3_-^15^N_1_]ALPAPIEK SIL Peptide synthesis*


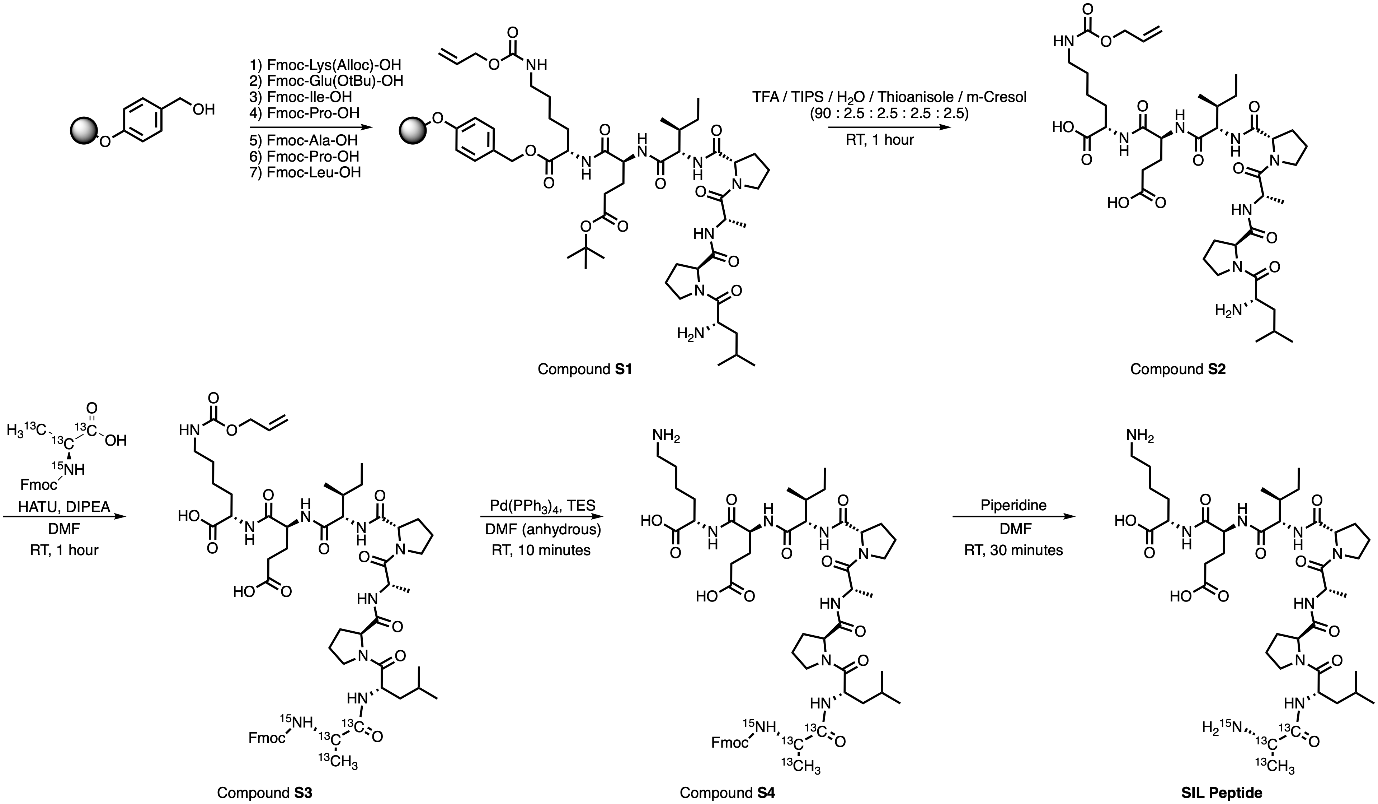


*General procedure for SPPS (GP1)*

Commercially available Wang resin (1 eq.) was swollen for 30 min in DMF (10 mL/g). A solution of amino acid (4 eq.), HATU (3.8 eq.), and DIPEA (8 eq.) in DMF (10 mL) was reacted for ten minutes and then added to the resin. The reaction was carried out at RT and quenched after three hours by washing the resin with DMF (5 times with 5 mL). The Fmoc protecting group was removed by incubating the resin with 10 mL of 20% v/v piperidine in DMF (3 times for 15 min), resin was then washed with DMF (5 times with 5 mL).

*Synthesis of compound S1*

For Compound S1, GP1 (1 gram scale, 1.1 moles) was sequentially iterated on Fmoc-Lys(Alloc), Fmoc-Glu(OTBu), Fmoc-Ile, Fmoc-Pro, Fmoc-Ala, Fmoc-Pro, Fmoc-Leu. (4.4 moles, 4 eq. for each step)

*Synthesis of compound S2*

Compound S1 was cleaved from the resin with a solution of 2.5% v/v TIPS, 2.5% v/v water, 2.5% v/v thioanisole, 2.5% v/v m-Cresol in Trifluoro Acetic Acid (TFA) (3 times for 1 h). In this step also the tert-butyl protecting group of the glutamic acid was removed. The solvent was evaporated on a rotavapor, the crude diluted 1:1 with DMF and purified by RP-HPLC on the Thermo Fisher UltiMate 3000. (224 mg, 264µmol, yield 24%)

**MS (ESI+)**, *m/z* calc for C_40_H_66_N_8_O_12_ [M+H]^+^ 851.5; observed 851.5

*Synthesis of compound S3*

^13^C_3_-^15^N_1_-Fmoc-Ala (10 mg, 31.7µmol, 1 eq.), HATU (12mg, 31.6 µmol, 1 eq.), and DIPEA (30 µL, 172 µmol, 5.4eq.) in DMF (100 µL) was reacted for 10 minutes. Compound S2 (27 mg, 31.7 µmol, 1eq) was added to the mixture and reaction left stirred for 50 min at RT. Ather this time the crude was purified by on the Agilent 1200 Series RP-HPLC. (14.6 mg, 12.7 µmol, yield 40%)

**MS (ESI+)**, *m/z* calc for C_58_H_81_N_9_O_15_ [M+H]^+^ 1148.6; observed 1148.5

*Synthesis of compound S4*

Compound S3 (14.6 mg, 12.7 µmol, 1eq) was dissolved in anhydrous DMF (200 µL). Pd(PPh_3_)_4_ (1.47 mg, 1.27 µmol, 0.1eq) and triethylsilane (14.8 mg, 127 µmol, 10eq) were added. Reaction left stirred for 10 minutes and subsequently quenched with water (600 µL). The crude mixture was filtered on celite and purified on the Agilent 1200 Series RP-HPLC (8.8 mg, 8.3 µmol, yield 65%)

**MS (ESI+)**, *m/z* calc for C_54_H_77_N_9_O_13_ [M+H]^+^ 1064.6; observed 1064.5

*Synthesis of SIL peptide*

Compound S4 (8.8 mg, 8.3 µmol, 1eq ) was dissolved in DMF (100 µL). Piperidine (3.3 µL, 33.2 µmol, 4 eq) was added and reaction left stirred for 30 minutes. The crude mixture was purified on the Agilent 1200 Series RP-HPLC and characterised by LC-HRMS (1.41 mg, 1.68 µmol, yield 20 %)

**MS (ESI+)**, *m/z* calc for C_39_H_67_N_9_O_11_ [M+2H]^++^ 421.75373; observed 421.75713 (8ppm accuracy)


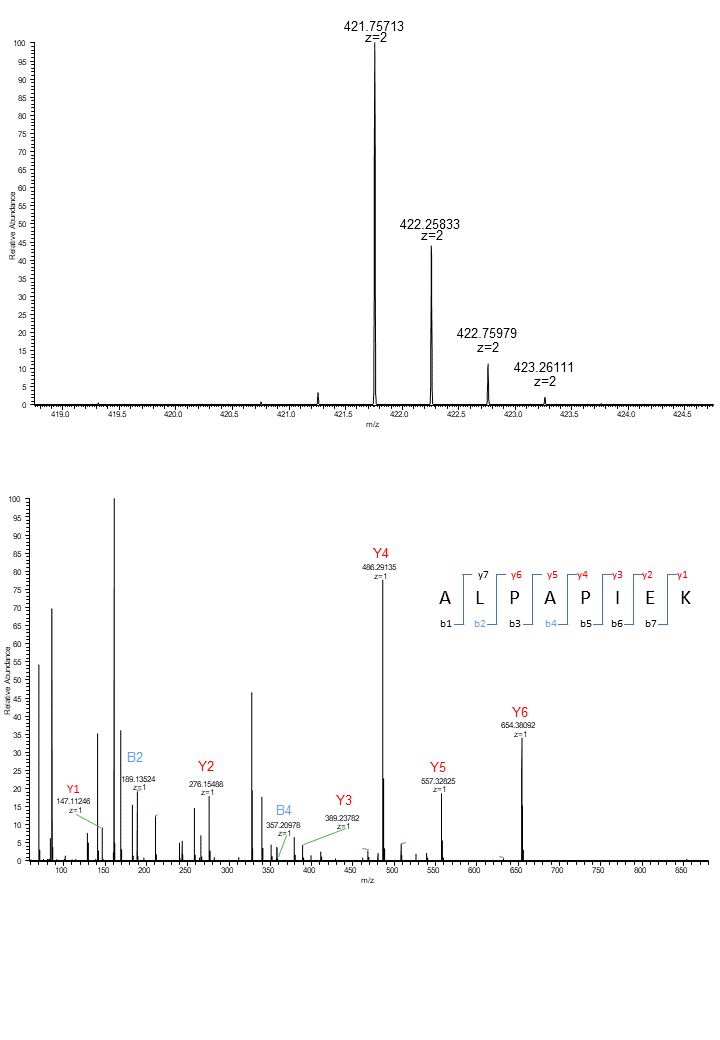


MS2 Spectra of SIL peptide***
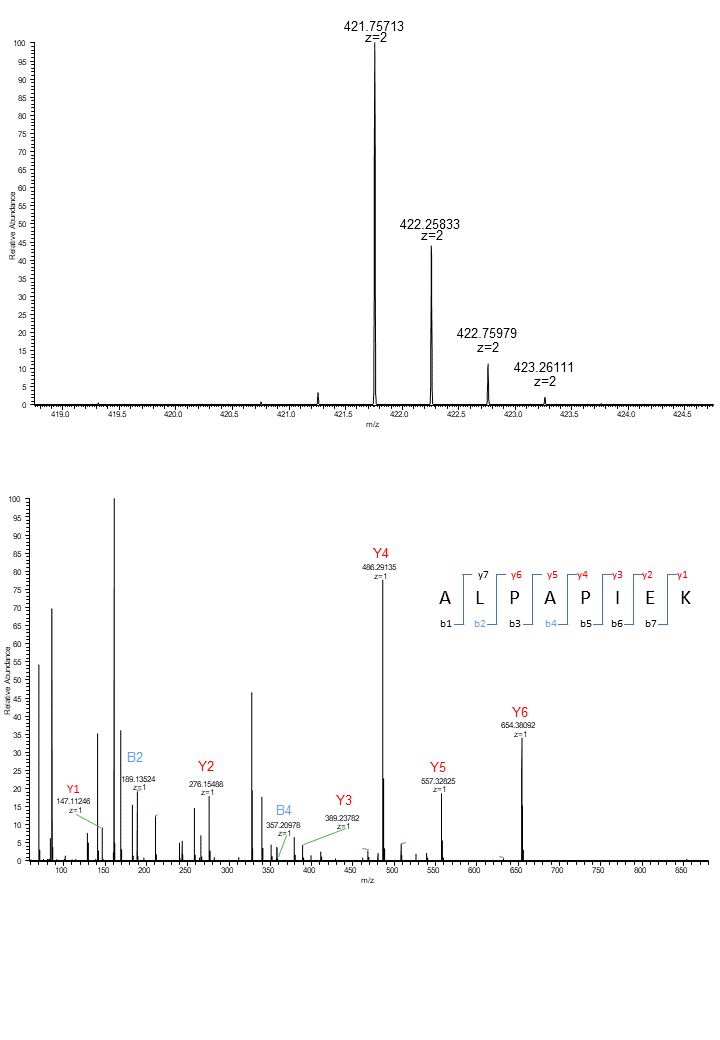
***

*Intact MS analysis*

600 ng of protein were subjected to HPLC-HRMS analysis on an Orbitrap Q-Exactive mass spectrometer (Thermo Fisher Scientific, RRID:SCR_020565) coupled to a Vanquish Flex LC system (Thermo Fisher Scientific) via an Ion Max-S API ESI source (Thermo Fisher, RRID:SCR_020148). Chromatographic separation was carried out at 80 °C on a MAbPac Reverse Phase column (100 mm x 2.1 mm, 4 µm particle size, 1500 Å pore size) using a 7.5-minute linear gradient from 10% to 70% of solvent B (0.1% formic acid in acetonitrile) at a flow rate of 600 µL/min. Ionization was carried out in positive Ion Mode, with 4 kV of spray voltage, 320 °C of capillary temperature, 90 S-lens RF level, 45 units of sheet gas, 15 units of Aux gas, 300 °C of Aux gas temperature, and 40 eV of in source CID. The detector was working in total ion scan with a scan range of 1000-6000 m/z, resolution of 17500 (FWHM at at 200 m/z), a maximum injection time of 200 ms, and 10 microscans. The resulting spectra were deconvoluted using Protein Deconvolution 4.0 software (Thermo Fisher;).

*Stable labelled isotopes Incorporation evaluation*

7.5 µg of SIL protein were diluted to a final volume of 200 µL with an aqueous solution containing Urea 1 M, 50 mM Tris-HCl, 1 mM CaCl_2_ pH 8.0. The protein was reduced with TCEP for 15 min at RT followed by 30 min at 65 ˚C and alkylated with Iodoacetamide for 30 mins in the dark. Digestion was achieved with trypsin (enzyme-protein ratio 1:50) at 37 ˚C overnight. After digestion, the sample was acidified with 10% formic acid and then subjected to C_18_ purification and desalting (Macro Spin Columns, Harvard Apparatus). The purified sample was dried under vacuum and resuspended in 30 µL of an aqueous solution containing 3% acetonitrile and 0.1% formic acid. 2 µL of the sample was then injected in the nanoLC-MS system. Chromatographic separation was carried out on an DNV PepMap Neo RSLC column (75 µm x 15 cm, particle size 2 µm, pore size 100 Å, Thermo Fisher Scientific) with a gradient program from 100% A (H_2_O, 0.1% FA), 0% B (ACN 0.1% FA) to 65% A, 35% B in 60 minutes on an Easy nanoLC 1000 (Thermo Fisher) at a flow rate of 300 nL/min. The LC system was coupled to a Q-Exactive mass spectrometer (Thermo Fisher) via a Nano Flex ion source (Thermo Fisher). Ionization was carried out with 2 kV of spray voltage, 250 °C of capillary temperature, 60 S Lens RF level. The mass spectrometer was working in a data-dependent top 10 acquisition mode with the following parameters: MS1 scan range: from 374.5 to 1425.5 m/z, HCD NCE: 27, Dynamic exclusion: 10 sec. MS/MS spectra were processed and analysed using Proteome Discoverer (PD, Thermo Fisher, Version 2.5, RRID:SCR_014477) and Skyline software (MacCoss Lab Software, version 22.2.0.527, RRID:SCR_014080). Database searches were performed against the antibody (i.e., L19 or F8) using Sequest as a search engine. Carbamidomethylation of cysteines was set as a fixed modification; oxidation of methionine, [^13^C_6_ ^15^N_2_] Lysine, and [^13^C_6_ ^15^N_4_] Arginine were set as variable modifications, and trypsin was set as cleavage specificity allowing a maximum of 2 missed cleavages. Data filtering was performed using percolator with a 1% False Discovery Rate (FDR). PD results were imported to Skyline, manually inspected and peaks area of heavy and light peptides manually integrated. Finally, SIL incorporation efficacy was calculated using the formula here reported: heavy peptide area / (heavy peptide area + light peptide area)

**Supplementary figures**

**
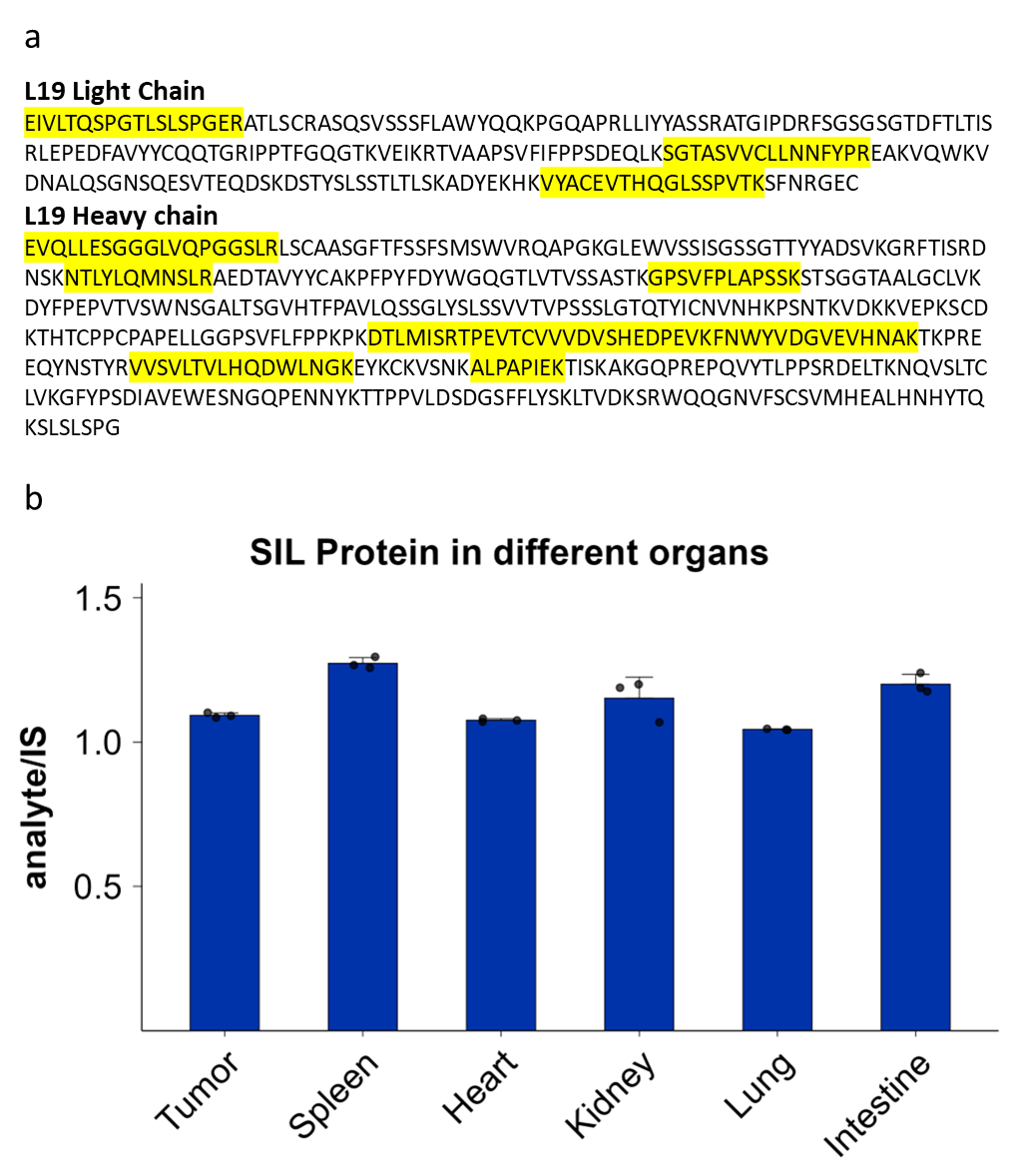
**

**Figure S1**: a) L19 IgG light chain and heavy chain protein sequence. Highlighted the sequence localization of the signature peptides selected for the absolute quantification b) Comparison of analyte to IS ratios measured in Tumor, Spleen, Heart, Kidney, Lung, and Intestine for the SIL Protein

**
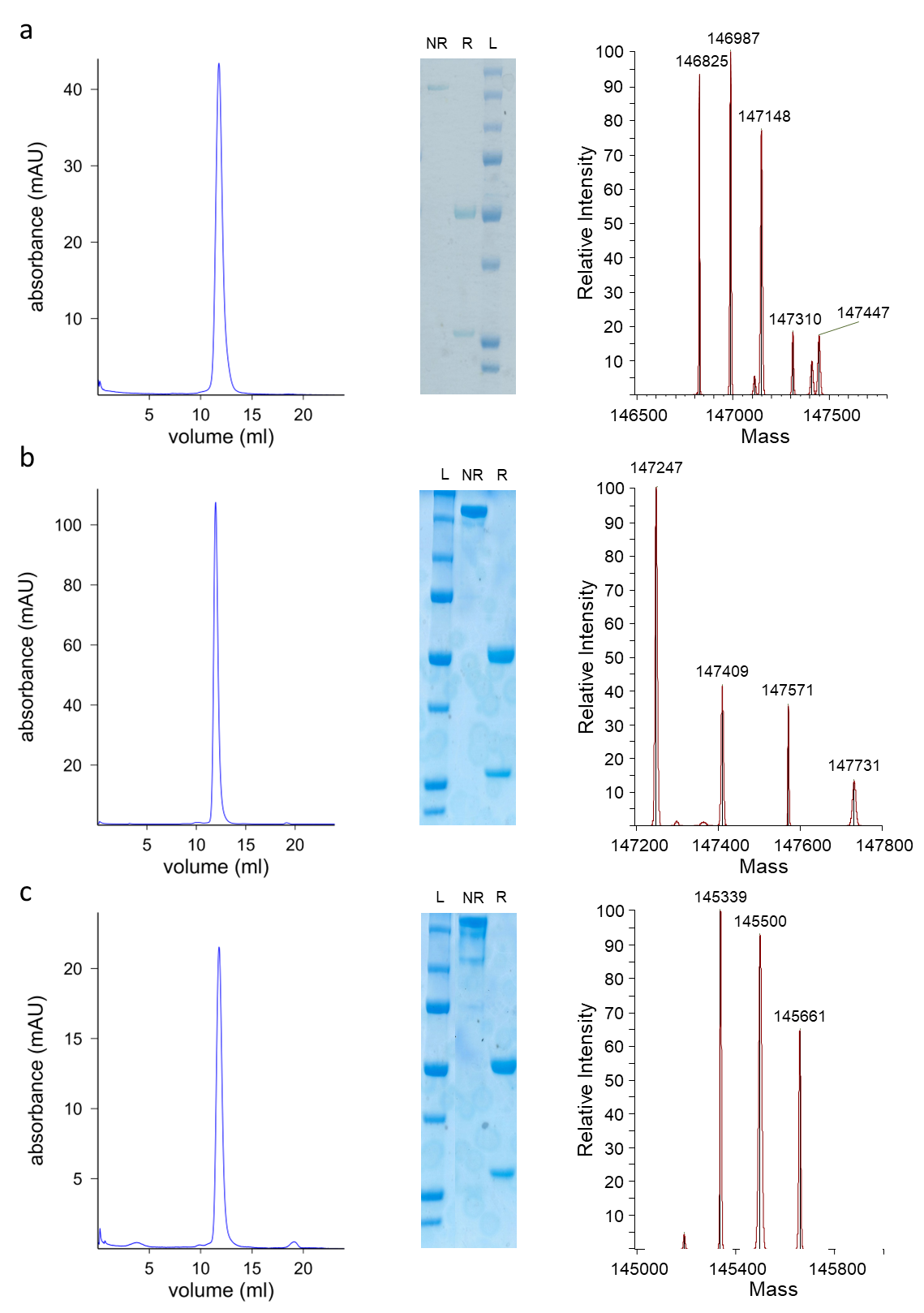
Figure S2**: From left to right SEC, SDS-PAGE and intact MS of A) L19 IgG B) F8 IgG and C) KSF IgG.


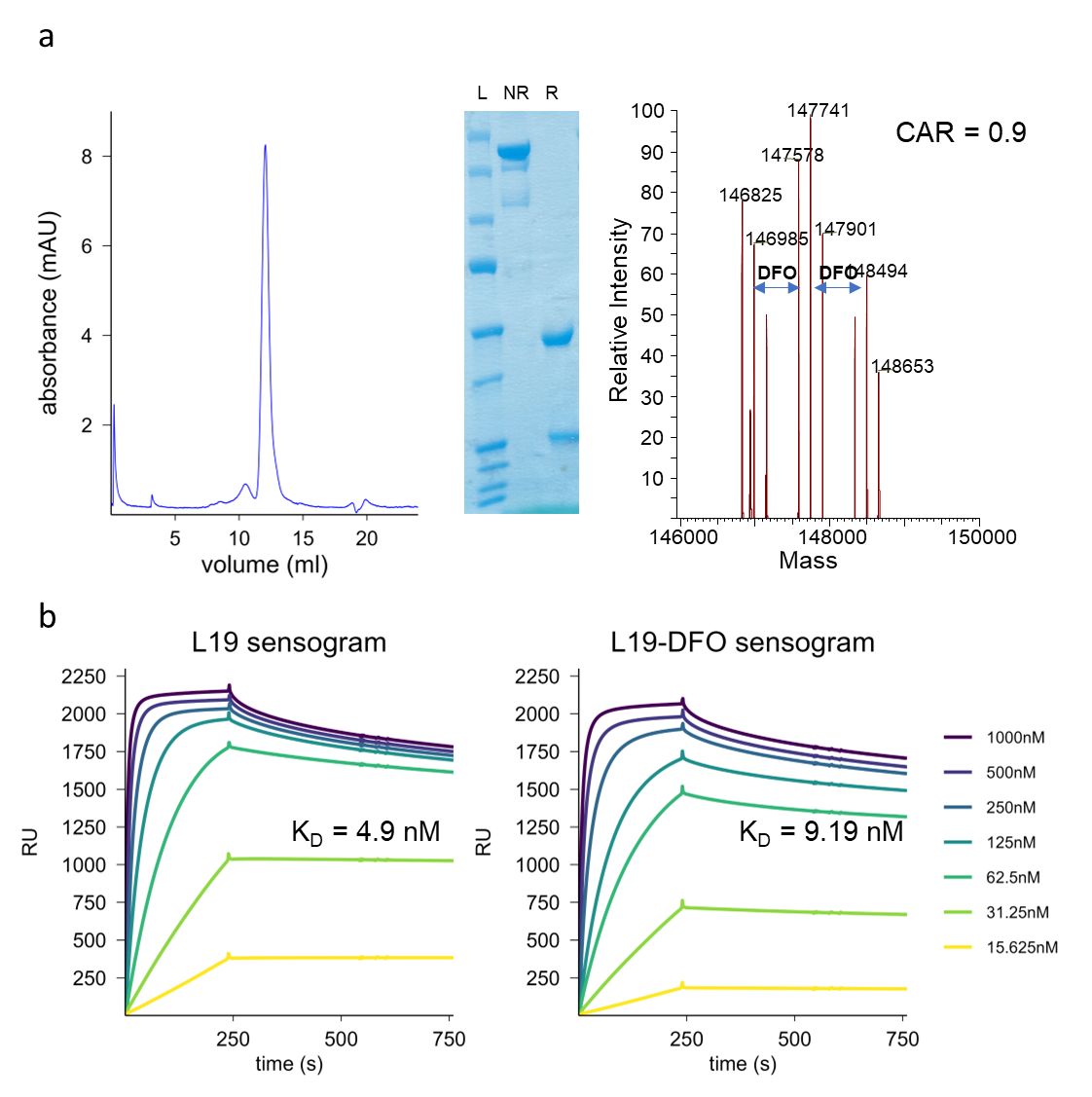


**Figure S3**: Quality Control of L19 IgG DFO. A) from left to right SEC, SDS-PAGE and intact MS. B) Biacore sensorgrams.

**
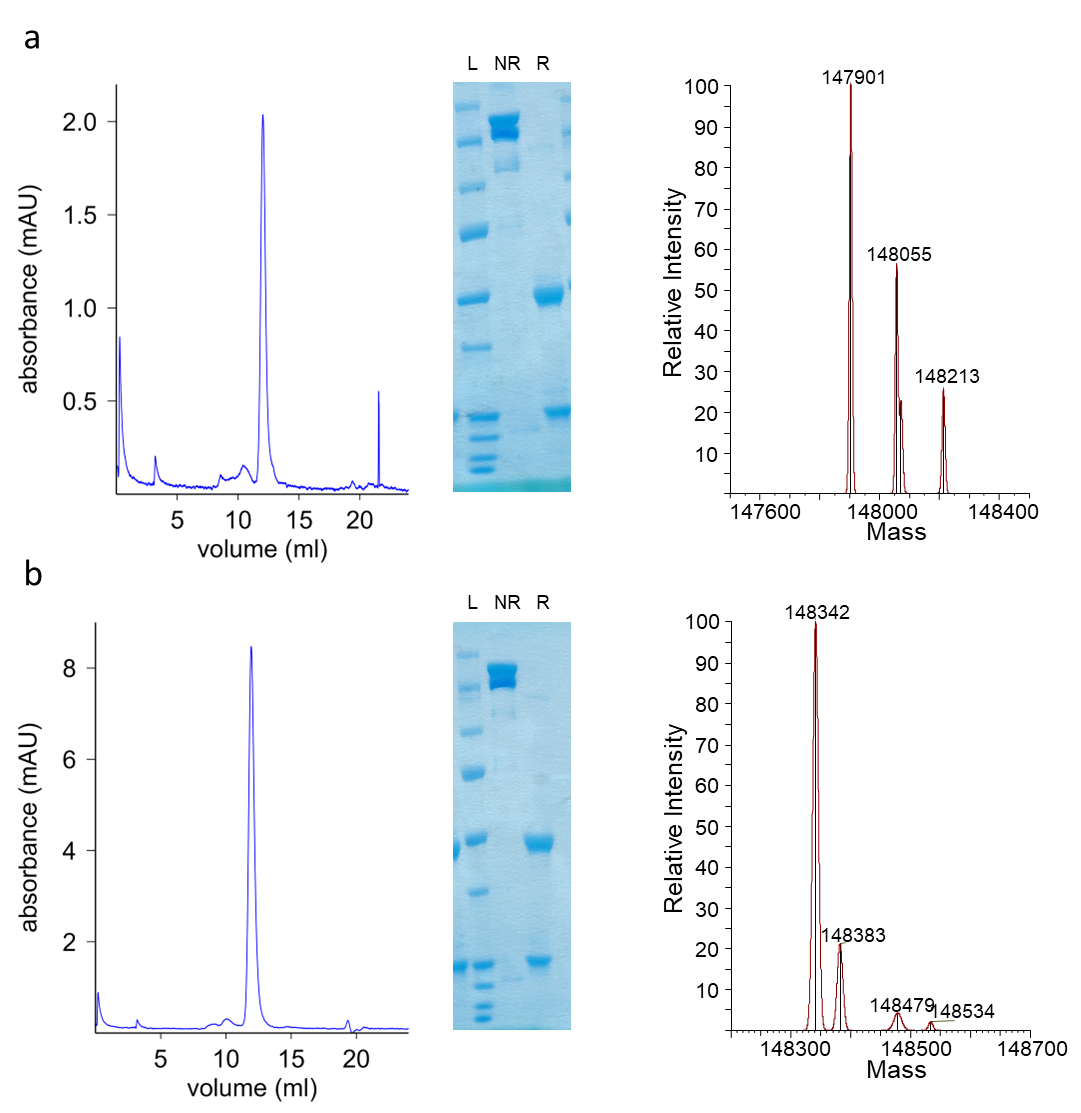
**

**Figure S4**: From left to right SEC, SDS-PAGE and intact MS of A) L19 IgG SILAC B) F8 IgG SILAC

**Supplementary tables**

**Table S1**: Comparison of analyte to IS ratios measured in blank matrices

| **Matrix** | **Internal Standard** | **Mean** | **SD** |
| --- | --- | --- | --- |
| Liver | SIL Protein | 0.98 | 0.00 |
| PBS | SIL Protein | 1.03 | 0.03 |
| Liver | SIL Peptide | 3.59 | 0.24 |
| PBS | SIL Peptide | 0.15 | 0.12 |
| Liver | Light Chain Isotype | 0.34 | 0.08 |
| PBS | Light Chain Isotype | 0.92 | 0.40 |
| Heart | SIL Protein | 1.08 | 0.01 |
| Intestine | SIL Protein | 1.10 | 0.00 |
| Kidney | SIL Protein | 1.40 | 0.52 |
| Lung | SIL Protein | 1.04 | 0.00 |
| Spleen | SIL Protein | 0.46 | 1.02 |
| Tumor | SIL Protein | 1.09 | 0.01 |

**Table S2**: L19 IgG signature peptides

| **Peptide** | **Charge state** | **% Heavy amino acid incorporation** |
| --- | --- | --- |
| ALPAPIEK | 2 | 99.66 |
| DTLMISR | 2 | 99.76 |
| EIVLTQSPGTLSLSPGER | 2 | 99.77 |
| EVQLLESGGGLVQPGGTSLR | 2 | 99.89 |
| FNWYVDGVEVHNAK | 2 | 99.55 |
|  | 3 | 99.62 |
| GPSVFPLAPSSK | 2 | 99.58 |
| NTLYLQMNSLR | 2 | 99.73 |
| SGTASVVCLLNNFYPR | 2 | 99.74 |
| TPEVTCVVVDVSHEDPEVK | 2 | 99.68 |
| VVSVLTVLHQDWLNGK | 2 | 99.63 |
|  | 3 | 99.62 |
| VYACEVTHQGLSSPVTK | 2 | 99.64 |

**Table S3**: F8 IgG signature peptides

| **Peptide** | **Charge state** | **% Heavy amino acid incorporation** |
| --- | --- | --- |
| ALPAPIEK | 2 | 99.73 |
| ASQSVSMPFLAWYQQKPGQAPR | 3 | 99.93 |
| DTLMISR | 2 | 99.76 |
| EIVLTQSPGTLSLSPGER | 2 | 99.76 |
|  | 3 | 99.83 |
| EVQLLESGGGLVQPGGSLR | 2 | 100.01 |
| FNWYVDGVEVHNAK | 3 | 99.70 |
| FSGSGSGTDFTLTISR | 2 | 99.78 |
| GPSVFPLAPSSK | 2 | 99.74 |
| LEPEDFAVYYCQQMR | 2 | 99.78 |
|  | 3 | 99.75 |
| SGTASVVCLLNNFYPR | 2 | 99.72 |
|  | 3 | 99.77 |
| STSGGTAALGCLVK | 2 | 99.70 |
| THTCPPCPAPELLGGPSVFLFPPKPK | 4 | 99.86 |
| TPEVTCVVVDVSHEDPEVK | 2 | 99.78 |
|  | 3 | 99.72 |
| TVAAPSVFIFPPSDEQLK | 2 | 99.69 |
|  | 3 | 99.75 |
| VVSVLTVLHQDWLNGK | 2 | 99.81 |
|  | 3 | 99.71 |
| VYACEVTHQGLSSPVTK | 2 | 99.62 |
|  | 3 | 99.69 |

**Table S4**: Mass Spectrometry-based *ex vivo* biodistribution of L19 IgG

| **Tissue** | **Mean** | **SD** |
| --- | --- | --- |
| Tumor | 26.55 | 6.65 |
| Plasma | 32.58 | 7.57 |
| Liver | 5.11 | 1.12 |
| Spleen | 5.03 | 0.88 |
| Heart | 5.52 | 2.16 |
| Kidney | 5.49 | 1.17 |
| Lung | 12.60 | 5.88 |
| Intestine | 4.52 | 1.69 |
| Stomach | 4.14 | 1.85 |

**Table S5**: Tumor to organ ratio MS based biodistribution of L19 IgG

| **Tissue** | **Tumor to organ ratio** |
| --- | --- |
| Plasma | 0.81 |
| Liver | 5.20 |
| Spleen | 5.28 |
| Heart | 4.81 |
| Kidney | 4.54 |
| Lung | 2.11 |
| Intestine | 5.87 |
| Stomach | 6.06 |

**Table S6**: Mass Spectrometry-based *ex vivo* biodistribution of F8 IgG

| **Tissue** | **Mean** | **SD** |
| --- | --- | --- |
| Tumor | 22.57 | 3.99 |
| Plasma | 29.62 | 7.52 |
| Liver | 5.95 | 1.23 |
| Spleen | 6.88 | 0.79 |
| Heart | 4.81 | 0.84 |
| Kidney | 12.12 | 1.96 |
| Lung | 18.24 | 3.87 |
| Intestine | 11.18 | 2.53 |
| Stomach | 10.49 | 2.48 |

**Table S7**: Tumor to organ ratio MS based biodistribution of F8 IgG

| **Tissue** | **Tumor to organ ratio** |
| --- | --- |
| Plasma | 0.76 |
| Liver | 3.79 |
| Spleen | 3.28 |
| Heart | 4.69 |
| Kidney | 1.86 |
| Lung | 1.24 |
| Intestine | 2.02 |
| Stomach | 2.15 |

**Table S8**: *Ex-vivo* radioactivity-based biodistribution of L19-^125^I

| **Tissue** | **Mean** | **SD** |
| --- | --- | --- |
| Tumor | 18.19 | 3.98 |
| Blood | 24.63 | 2.91 |
| Liver | 6.10 | 1.19 |
| Spleen | 6.21 | 0.86 |
| Heart | 7.94 | 0.65 |
| Kidney | 8.91 | 1.53 |
| Lung | 14.74 | 3.86 |
| Intestine | 6.03 | 0.66 |
| Stomach | 5.09 | 1.09 |

**Table S9**: *Ex-vivo* radioactivity-based biodistribution of L19-DFO-^89^Zr

| **Tissue** | **Mean** | **SD** |
| --- | --- | --- |
| Tumor | 17.66 | 3.26 |
| Blood | 19.34 | 3.47 |
| Liver | 7.94 | 1.55 |
| Spleen | 8.34 | 2.93 |
| Heart | 7.00 | 1.89 |
| Kidney | 8.44 | 1.42 |
| Lung | 10.22 | 3.02 |
| Intestine | 5.77 | 1.44 |
| Stomach | 4.35 | 1.59 |

**Table S10**: *Ex-vivo* Mass Spectrometry-based biodistribution of decayed L19-DFO-^89^Zr

| **Tissue** | **Mean** | **SD** |
| --- | --- | --- |
| Tumor | 16.66 | 4.60 |
| Plasma | 20.01 | 0.42 |
| Liver | 4.76 | 1.21 |
| Heart | 4.52 | 1.85 |
| Kidney | 7.10 | 1.62 |
| Lung | 13.88 | 2.16 |

**Table S11**: Multiple t-test comparison of mass spectrometry and radioactivity biodistribution of L19-DFO-^89^Zr

| **Tissue** | **p-value** |
| --- | --- |
| Tumor | 0.735 |
| Plasma | 0.810 |
| Liver | 0.018 |
| Heart | 0.110 |
| Kidney | 0.263 |
| Lung | 0.096 |

**Antibodies Sequence**

*L19 IgG*

*Light Chain*

EIVLTQSPGTLSLSPGERATLSCRASQSVSSSFLAWYQQKPGQAPRLLIYYASSRATGIPDRFSGSGSGTDFTLTISRLEPEDFAVYYCQQTGRIPPTFGQGTKVEIKRTVAAPSVFIFPPSDEQLKSGTASVVCLLNNFYPREAKVQWKVDNALQSGNSQESVTEQDSKDSTYSLSSTLTLSKADYEKHKVYACEVTHQGLSSPVTKSFNRGEC

*Heavy Chain*

EVQLLESGGGLVQPGGSLRLSCAASGFTFSSFSMSWVRQAPGKGLEWVSSISGSSGTTYYADSVKGRFTISRDNSKNTLYLQMNSLRAEDTAVYYCAKPFPYFDYWGQGTLVTVSSASTKGPSVFPLAPSSKSTSGGTAALGCLVKDYFPEPVTVSWNSGALTSGVHTFPAVLQSSGLYSLSSVVTVPSSSLGTQTYICNVNHKPSNTKVDKKVEPKSCDKTHTCPPCPAPELLGGPSVFLFPPKPKDTLMISRTPEVTCVVVDVSHEDPEVKFNWYVDGVEVHNAKTKPREEQYNSTYRVVSVLTVLHQDWLNGKEYKCKVSNKALPAPIEKTISKAKGQPREPQVYTLPPSRDELTKNQVSLTCLVKGFYPSDIAVEWESNGQPENNYKTTPPVLDSDGSFFLYSKLTVDKSRWQQGNVFSCSVMHEALHNHYTQKSLSLSPG

F8 IgG

*Light Chain*

EIVLTQSPGTLSLSPGERATLSCRASQSVSMPFLAWYQQKPGQAPRLLIYGASSRATGIPDRFSGSGSGTDFTLTISRLEPEDFAVYYCQQMRGRPPTFGQGTKVEIKRTVAAPSVFIFPPSDEQLKSGTASVVCLLNNFYPREAKVQWKVDNALQSGNSQESVTEQDSKDSTYSLSSTLTLSKADYEKHKVYACEVTHQGLSSPVTKSFNRGEC

*Heavy Chain*

EVQLLESGGGLVQPGGSLRLSCAASGFTFSLFTMSWVRQAPGKGLEWVSAISGSGGSTYYADSVKGRFTISRDNSKNTLYLQMNSLRAEDTAVYYCAKSTHLYLFDYWGQGTLVTVSSASTKGPSVFPLAPSSKSTSGGTAALGCLVKDYFPEPVTVSWNSGALTSGVHTFPAVLQSSGLYSLSSVVTVPSSSLGTQTYICNVNHKPSNTKVDKKVEPKSCDKTHTCPPCPAPELLGGPSVFLFPPKPKDTLMISRTPEVTCVVVDVSHEDPEVKFNWYVDGVEVHNAKTKPREEQYNSTYRVVSVLTVLHQDWLNGKEYKCKVSNKALPAPIEKTISKAKGQPREPQVYTLPPSRDELTKNQVSLTCLVKGFYPSDIAVEWESNGQPENNYKTTPPVLDSDGSFFLYSKLTVDKSRWQQGNVFSCSVMHEALHNHYTQKSLSLSPG

KSF IgG

*Light Chain*

SSELTQDPAVSVALGQTVRITCQGDSLRSYYASWYQQKPGQAPVLVIYGKNNRPSGIPDRFSGSSSGNTASLTITGAQAEDEADYYCNSSPLNRLAVVFGGGTKLTVLGQPKAAPSVTLFPPSSEELQANKATLVCLISDFYPGAVTVAWKADSSPVKAGVETTTPSKQSNNKYAASSYLSLTPEQWKSHKSYSCQVTHEGSTVEKTVAPTECS

*Heavy Chain*

EVQLLESGGGLVQPGGSLRLSCAASGFTFSSYAMSWVRQAPGKGLEWVSAISGSGGSTYYADSVKGRFTISRDNSKNTLYLQMNSLRAEDTAVYYCAKSPKVSLFDYWGQGTLVTVSSASTKGPSVFPLAPSSKSTSGGTAALGCLVKDYFPEPVTVSWNSGALTSGVHTFPAVLQSSGLYSLSSVVTVPSSSLGTQTYICNVNHKPSNTKVDKKVEPKSCDKTHTCPPCPAPELLGGPSVFLFPPKPKDTLMISRTPEVTCVVVDVSHEDPEVKFNWYVDGVEVHNAKTKPREEQYNSTYRVVSVLTVLHQDWLNGKEYKCKVSNKALPAPIEKTISKAKGQPREPQVYTLPPSRDELTKNQVSLTCLVKGFYPSDIAVEWESNGQPENNYKTTPPVLDSDGSFFLYSKLTVDKSRWQQGNVFSCSVMHEALHNHYTQKSLSLSPGK
